# Impact of rs1805127 and rs55742440 Variants on Atrial Remodeling in Hypertrophic Cardiomyopathy Patients with Atrial Fibrillation: A Romanian Cohort Study

**DOI:** 10.3390/ijms242417244

**Published:** 2023-12-08

**Authors:** Nicoleta-Monica Popa-Fotea, Nicoleta Oprescu, Alexandru Scafa-Udriste, Miruna Mihaela Micheu

**Affiliations:** 1 Department 4—Cardio-Thoracic Pathology, University of Medicine and Pharmacy Carol Davila, Eroii Sanitari Bvd. 8, 050474 Bucharest, Romania; fotea.nicoleta@yahoo.com; 2 Department of Cardiology, Clinical Emergency Hospital of Bucharest, Calea Floreasca 8, 014461 Bucharest, Romania; nicoleta_m_oprescu@yahoo.com

**Keywords:** atrial fibrillation, genetic polymorphism, variant, next generation sequencing, hypertrophic cardiomyopathy

## Abstract

Atrial fibrillation (AFib) is characterized by a complex genetic component. We aimed to investigate the association between variations in genes related to cardiac ion handling and AFib in a cohort of Romanian patients with hypertrophic cardiomyopathy (HCM). Forty-five unrelated probands with HCM were genotyped by targeted next-generation sequencing (NGS) for 24 genes associated with cardiac ion homeostasis. Subsequently, the study cohort was divided into two groups based on the presence (AFib+) or absence (AFiB−) of AFib detected during ECG monitoring. We identified two polymorphisms (rs1805127 located in *KCNE1* and rs55742440 located in *SCN1B*) linked to AFib susceptibility. In AFib+, rs1805127 was associated with increased indexed left atrial (LA) maximal volume (LAVmax) (58.42 ± 21 mL/m^2^ vs. 32.54 ± 6.47 mL/m^2^, *p* < 0.001) and impaired LA strain reservoir (LASr) (13.3 ± 7.5% vs. 24.4 ± 6.8%, *p* < 0.05) compared to those without respective variants. The rs55742440 allele was less frequent in patients with AFib+ (12 out of 25, 48%) compared to those without arrhythmia (15 out of 20, 75%, *p* = 0.05). Also, AFib+ rs55742440 carriers had significantly lower LAVmax compared to those who were genotype negative. Among patients with HCM and AFib+, the rs1805127 variant was accompanied by pronounced LA remodeling, whereas rs55742440’s presence was related to a milder LA enlargement.

## 1. Introduction

AFib is the most common cardiac arrhythmia in adults, imposing a considerable burden on both patients and healthcare providers due to substantial morbidity and mortality [1]. Globally, its prevalence is estimated to be approximately 60 million cases, resulting in over 8 million disability-adjusted life years. The estimated lifetime risk of developing AFib is around 33%, although it may vary based on patient-specific factors such as age, sex, race, and comorbidities [2]. Recently, there has been a growing recognition of AFib as a multifaceted and dynamic condition, which is characterized by a substantial genetic foundation and complex epigenetic mechanisms [3,4,5,6]. Existing evidence suggests that both common and rare genetic variants contribute to increased susceptibility to AFib especially when combined with ethnic-specific risk factors [7]. These findings carry significant clinical implications, including the potential to guide the antiarrhythmic treatments and to identify appropriate candidates for ablation therapy based on genetic background. Moreover, the incorporation of common genetic variants into AFib risk scores has demonstrated enhanced predictive accuracy and improved risk stratification [8,9]. Structural and functional alterations in atrial tissue, involving electrical, mechanical, and structural remodeling processes, contribute to AFib appearance and persistence. Electrical remodeling leads to changes in ion channel function, affecting the cardiac action potential duration and refractoriness, thereby facilitating AFib perpetuation. Mechanical remodeling, encompassing atrial dilation, fibrosis, and hypertrophy, is also crucial. Atrial dilation occurs due to increased pressure or volume overload, stretching the atrial walls. Fibrosis, characterized by excessive collagen deposition, disrupts electrical conduction pathways, and hypertrophy increases electrical and mechanical disturbances. Additionally, neurohormonal and inflammatory factors can promote atrial electrical instability and remodeling by modulating ion channel function, calcium handling, and fibrotic processes, exacerbating the arrhythmia [3,10,11]. Many of these abnormalities are also hallmark features of HCM [12]; hence, AFib is four to six times more common in HCM individuals than in the general population [13]. The presence of HCM does not necessarily entail the occurrence of AFib; nevertheless, genetic variants have the potential to modulate AFib vulnerability by modifying the structure of proteins involved in the aforesaid cellular processes. Lee et al. reported that in patients with *MYH7* mutations, there was a higher incidence of AFib independently of other clinical and echocardiographic factors compared with other sarcomeric genes mutations [14]. Candidate genome-wide association studies have identified many genes possibly involved in the development of atrial fibrillation, but the genotype dependency of AFib in HCM is less studied; for example, in the case of cardiac Troponin T gene mutations, the association with AFib is dependent on the pathophysiological mechanism induced by the variant [15]. Consequently, different mutations in the same gene have different impacts on the atrial arrhythmia appearance. In this study, we focused on the association between variations in genes related to cardiac ion handling and AFib in a cohort of adult patients with HCM.

## 2. Results

### 2.1. Study Population and Echocardiography Parameters

The study cohort consisted of 25 patients who had both HCM and AFib (AFib+ group), as identified through previous ECG monitoring. None of these individuals were experiencing permanent atrial fibrillation. Additionally, there were 20 HCM subjects without detected arrhythmia (AFib− group). Table 1 provides a summary of the demographic and conventional echocardiographic data for these two clusters. All included subjects were Caucasians. The mean age of AFib+ group was 51.67 ± 14.55 years old and 80% were males, while the mean age of AFib− was 46.35 ± 15 years with 75% males. There were no significant differences in age, gender, history of sudden cardiac death (SCD), or implantable cardiac defibrillator (ICD) presence between the two groups. There was an important difference in the risk of SCD at 5 years, patients with HCM and AFib+ displaying a higher risk (5.49 ± 3.92%) compared with those with HCM and AFib− (2.83 ± 1.37%), *p* = 0.044. The two groups did not differ significantly in terms of LV maximal wall thickness, LVEF, LVEDD, LVESD, LVEDV and LVESV (Table 1). Patients with AFib+ had increased LAVmax and LAVpre-A compared with those without AFib. LATEF was decreased in the AFib+ group (54 ± 10.3%) in contrast with the AFib− group (60 ± 8.8%) (*p* = 0.041). The dysfunction of LA reservoir function was also confirmed by LA deformation analysis depicting reduced LASr (21 ± 5.6%) and pLASRr (0.8 ± 0.4 s^−1^) in subjects with AFib+ compared with AFib− (LASr = 25 ± 4%, *p* = 0.008 and pLASRr = 1.2 ± 0.67 s^−1^, *p* = 0.025).

### 2.2. Genes and Variants

Of the 174 genes covered by the TruSight Cardio Sequencing Kit, only 24 genes were considered in this analysis, specifically those related to cardiac ion homeostasis. The mean depth of sequence coverage across target regions was 177×. The complete list of analyzed genes and encoded proteins is depicted in Table 2. Table 3 details the pathogenic or likely pathogenic variants in sarcomeric genes identified in the cohort. Also, comprehensive data regarding all the variants identified is provided, detailing information such as genotype, rs ID, allele frequency in the gnomAD v4 database, published association with AFib, and in how many AFib+ patients these variants were found (Supplementary Table S1).

#### 2.2.1. Analysis of LA Traits and Potassium Channel Variants

There were no statistically significant differences in the number of potassium channel variants identified between the two groups with a mean of 2.56 ± 0.76 variants in AFib+ and 2.8 ± 0.89 variants in AFib− (*p* = 0.604) (Table 4).

There was no correlation observed between the number of variants in potassium channels and the size or function of the LA within AFib+ patients. We did not observe any differences between the two groups regarding c.112A>G polymorphism (reference SNP ID: rs1805127); the variant was detected in 80% of AFib+ cases (20 out 25) and in all AFib− individuals (N = 20). But subjects with AFib+ and the c.112A>G polymorphism of *KCNE1* had significantly larger LAVmax (58.42 ± 21 mL/m^2^) compared to those without respective polymorphism (32.54 ± 6.47 mL/m^2^, *p* < 0.001). Moreover, those with AFib+ and c.112A>G polymorphism had an impaired reservoir function (LASr = 13.3 ± 7.5%) compared to those with AFib+ but without the variant (LASr = 24.4 ± 6.8%, *p* < 0.05), conduit and pump function being similar regardless of the presence of genetic variants in *KCNE1* gene. No additional correlations were found between other potassium channel-coding genes variants and LA traits.

#### 2.2.2. Analysis of LA Traits and Sodium Channel Variants

There were no statistically significant differences in the number of sodium channel variants between the two subgroups: AFib+ (1.92 ± 1.41) or AFib− (2 ± 1.62 variants, *p* = 0.078) (Table 5).

Patients with AFib+ and two or more sodium channel variants (n = 14) had significantly larger LAVmax (86.4 ± 23.9 mL/m^2^) compared to those with one or no variants (n = 11) (65.34 ± 10.7 mL/m^2^, *p* = 0.023). The same observation remains valid after excluding Na channel variants with MAF < 1%; subjects with AFib+ and two or more variants in sodium channels (n = 13) had significantly higher LAVmax (62.9 ± 8.4 mL/m^2^) compared with those having one or no variant (n = 10) (54.32 ± 9 mL/m^2^), *p* = 0.024.

The *SCN1B* c.629T>C polymorphism (reference SNP ID: rs55742440, protein sequence: p.Leu210Pro) was found less frequently amid patients with AFib+ (N = 12/25, 48%) compared to AFib− (N = 15/20, 75%, *p* = 0.05). Moreover, AFib+ subjects harboring the c.629T>C variant had significantly lower LAVmax (24.7 ± 5.4 mL/m^2^) compared to those without this polymorphism (64.67 ± 9.29 mL/m^2^, *p* < 0.001). There were no additional significant correlations between other sodium channel-coding gene variants and other LA traits.

#### 2.2.3. Analysis of LA Traits and Calcium Channel Variants

There were no statistically significant differences concerning the number of calcium channel variants between the two subgroups: AFib+ (a mean number of 5 ± 1.67) or AFib− (a mean number of 4.45 ± 1.19), *p* = 0.318 (Table 6). Within AFib+, no correlation was identified between the number of Ca channel variants or specific variants and LA volumes and/or LA mechanics.

## 3. Discussion

In this study, we investigated the influence of low-frequency and common variants within specific genes on the occurrence of AFib in a cohort of adult patients with HCM. For our analysis, we selected a list of 24 genes (Table 2) involved in ion homeostasis within cardiac cells, for which literature data have shown an association with AFib [3,5,7].

The overall demographic profile of our study group was previously documented [37]; the average age at enrollment was approximately in the fifth decade of life, with a higher proportion of male participants, analogous to findings obtained from the Romanian Registry of Hypertrophic Cardiomyopathy [38]. As showed in Table 1, there were no significant differences between the AFib+ (N = 25) and AFib− (N = 20) groups in terms of age, gender distribution, history of sudden cardiac death (SCD), presence of implantable cardiac defibrillators (ICD), and LV size and function. Predictably, we noticed a significant difference in the risk of SCD at 5 years between the two groups. Patients with HCM and AFib+ displayed an almost twofold higher risk of SCD compared to AFib− (5.49 ± 3.92% vs. 2.83 ± 1.37%). Comparable statistics were detected in a nationwide study conducted in Taiwan; after adjustment for various confounders (such as age, sex, comorbidities and medications), the presence of AFib was linked to a threefold increase in the risk of SCD, demonstrating its substantial impact on adverse cardiac events in HCM patients [39].

LA enlargement has been commonly recognized as a marker of increased risk for cardiovascular events and adverse outcomes in various cardiac conditions, including HCM [40,41]. Our data showed a notable difference between groups regarding both LA size and its function. Similar to evidence from large cohorts [42,43,44,45], AFib+ was associated with augmented LA volumes and reduced atrial performance. Specifically, patients with AFib exhibited increased LAVmax and LAVpre-A compared to AFib−. Furthermore, LATEF was found to be decreased in the AFib+ group (54 ± 10.3%) compared to the AFib− group (60 ± 8.8%, *p* = 0.041). Additionally, the dysfunction of LA’s reservoir function was confirmed through analysis of LA deformation. The findings revealed reduced LASr (21 ± 5.6%) and pLASRr (0.8 ± 0.4 s^−1^) in subjects with AFib+ compared to AFib−. Explicitly, LASr was 25 ± 4% in the AFib− group (*p* = 0.008) and pLASRr was 1.2 ± 0.67 s^−1^ (*p* = 0.025). The reduction in reservoir function was most likely due to the reduction in LA compliance, which was indirectly evaluated by measuring LASI. LASI showed a significant increase in the AFib+ group (1.8 ± 0.4) compared to the AFib− group (1.4 ± 0.8).

### 3.1. Analysis of LA Traits and Potassium Channel Variants

Potassium channels play a crucial role in cardiac electrical activity, and genetic variants in potassium channel genes can impact atrial repolarization and electrical stability, potentially increasing the risk of AFib [16,22,46]. In our study, there were no statistically significant differences found in the number of K channel variants identified between the two groups. The mean number of variants was 2.56 ± 0.76 in AFib+ and 2.8 ± 0.89 in AFib− (*p* = 0.604). Also, there was no correlation observed between the number of variants in potassium channels and the size or function of the LA within AFib+ patients.

Upon analyzing each detected potassium channel variant individually in relation to AFib, only a single one showed a potential relationship with the condition, namely c.112A>G polymorphism in the *KCNE1* gene. The *KCNE1* gene encodes the β-subunit of voltage-gated potassium channel Kv7.1, producing the slow delayed rectifier potassium current (IKs). The c.112A>G polymorphism results in the substitution of serine to glycine at position 38 (p.Ser38Gly) in the encoded protein.

Within the AFib+ group, we observed that c.112A>G positive subjects exhibited greater LA enlargement (LAVmax 58.42 ± 21 mL/m^2^) compared to those without respective polymorphism (LAVmax 32.54 ± 6.47 mL/m^2^, *p* < 0.001). Moreover, the presence of the aforesaid SNP was associated with impaired reservoir function (LASr = 13.3 ± 7.5%) compared to those lacking the variant (LASr = 24.4 ± 6.8%, *p* < 0.05). However, conduit and pump function remained similar regardless of the presence of genetic variants in the *KCNE1* gene. Existing evidence advocate that AFib causally increases LA size and decreases its function [47,48,49]; at the same time, atrial remodeling enhances the vulnerability to AFib occurrence and persistence [42,50,51,52], thus perpetuating the cycle. So, we could hypothesize that patients harboring rs1805127 might carry increased AFib burden compared with those lacking it, and they also are more prone to recidivate. Higher AFib burden is associated with higher cardiovascular morbidity and mortality [53,54], so an early identification of high-risk patients is of paramount importance.

Several studies have investigated the relationship between the c.112A>G polymorphism and AFib susceptibility, but the results have been conflicting. While certain findings indicate a potential correlation between this particular genetic variation and an elevated risk of AFib [55,56,57], other studies have not found supporting evidence for such an association [58,59]. It is important to note that the exact contribution of rs1805127 to AFib risk may vary depending on the population studied and other genetic and environmental modifiers. This variability has been demonstrated by Liang et al. in their meta-analysis, which encompassed ten case-control studies involving 2099 cases and 2252 controls [60]. The overall analysis exposed that the rs1805127 polymorphism was linked to an elevated risk of AFib across all four comparisons: homozygote comparison (GG vs. AA), heterozygote comparison (GA vs. AA), dominant model (GA/GG vs. AA), and recessive model (GG vs. GA/AA). Further subgroup analyses based on ethnicity revealed that the risk remained consistent in all four comparisons among the Asian population. However, among the Caucasian population, the heightened risk of AFib was observed in the heterozygote comparison and dominant model, while no significant association was found in the homozygote comparison and recessive model.

We did not observe any differences between the groups regarding the presence of c.112A>G. Although no significant difference was found concerning the distribution of c.112A>G, the association between this polymorphism and more pronounced LA remodeling may be due to the fact that the mentioned variant is a modifier variant that only contributes to the atrial phenotype in conjunction with other genetic variants. Our sample size was too small to enable specific analysis depending on the presence of the allele in a homozygous or heterozygous state.

### 3.2. Analysis of LA Traits and Sodium Channel Variants

The association between sodium channel variants and AFib has been extensively investigated; findings revealed various alleles that are linked either to an increased susceptibility to AFib [61,62,63] or to potential protection against it [64].

In our research, the analysis of sodium channel variants did not reveal significant differences concerning the number of mutations between patients AFib+ and AFib− (1.92 ± 1.41 vs. 2 ± 1.62 variants, *p* = 0.078).

In the cohort AFib+, those who possessed two or more sodium channel variants (n = 14) demonstrated a statistically significant increase in LAVmax compared to subjects with either one or no variants (n = 11) (86.4 ± 23.9 mL/m^2^ vs. 65.34 ± 10.7 mL/m^2^, *p* = 0.023). This finding remained consistent even after excluding sodium channel variants with AF below 1% (62.9 ± 8.4 mL/m^2^ vs. 54.32 ± 9 mL/m^2^, *p* = 0.024), suggesting that the number of mutations harbored by an individual may play a role not only in specific mutations but also in the pathogenesis of AFib and LA remodeling. Indeed, prior evidence suggests that the effect is dose-dependent [64].

We observed a negative relationship between *SCN1B* c.629T>C polymorphism and AFib+. The *SCN1B* gene encodes the β-subunit of voltage-gated sodium channel Nav1.5. The respective SNP exhibited a lower prevalence among patients AFib+ (N = 12/25, 48%) in contrast to AFib− (N = 15/20, 75%, *p* = 0.05). Furthermore, within the AFib+ group, subjects possessing the aforesaid variant demonstrated markedly reduced LA volumes (24.7 ± 5.4 mL/m^2^) compared to individuals lacking it (64.67 ± 9.29 mL/m^2^, *p* < 0.001). Accordingly, one can postulate that rs55742440 genotype-positive subjects may exhibit a reduced AFib burden compared with those genotype-negative and/or might be less likely to experience AFib recurrence.

### 3.3. Strength and Limitations

To our knowledge, this study is the first of its kind to investigate the association between genetic variants and AFib in a Romanian cohort. By exploring this link in a specific population, it provides valuable insights into the genetic basis of AFib within that population. This contributes to the understanding of AFib’s genetic heterogeneity and may have implications for personalized medicine and targeted therapies in the Romanian population.

We are aware of some limitations that should be taken into consideration. Firstly, the cohort size is small, and the study was monocentric, which may affect the generalizability of the findings to larger populations, from this deriving the need for a replication study based on a larger population. Secondly, the two studied SNPs were not confirmed by Sanger sequencing. Thirdly, the lack of follow-up for AFib limits the ability to assess the long-term implications of rs1805127 and rs55742440 polymorphism. Fourthly, the analysis was exploratory in nature and did not consider the possibility that an association may be caused by multiple hypothesis testing. Furthermore, the analysis was focused solely on exonic variants, potentially overlooking other non-exonic genetic variations that could contribute to AFib. Therefore, further studies with larger cohorts, longitudinal follow-up, and broader genetic analyses would be valuable in confirming and expanding upon these findings.

## 4. Materials and Methods

### 4.1. Study Population

We have analyzed retrospectively 45 Caucasians subjects diagnosed with HCM according to the criteria of the European Society of Cardiology [65]. The diagnosis was established after a comprehensive evaluation based on clinical, imaging and genetic criteria. The study was approved by the ethical committee of Emergency Clinical Hospital, Bucharest, Romania, and inclusion in the study was preceded by the signature of a written informed consent. The cohort was further on divided into two subgroups depending on the detection or absence of AFib at the time of inclusion or at previous 24 h ECG monitoring.

### 4.2. Genetic Testing

The genetic testing methodology has been previously described in detail [37,66]. In summary, patients’ DNA was extracted from blood using a MagCore Genomic DNA Whole Blood Kit (RBC Bioscience, Taipei, Taiwan) and subsequently quantified using a Qubit dsDNA HS assay kit (Life Technologies, Carlsbad, CA, USA). Targeted next-generation sequencing (NGS) was performed on a MiSeq platform with the 150 bp paired-end protocol (Illumina, San Diego, CA, USA) with a TruSight Cardio Sequencing Kit (Illumina, San Diego, CA, USA). An initial amount of 50 ng of genomic DNA was used for optimal gene enrichment.

### 4.3. Variant Assessment

Sequencing data were analyzed using the MiSeq Reporter software v2.4 (Illumina, San Diego, CA, USA), adopting hg19/GRCh37 as a genome reference. To interrogate genomic variants, we used VariantStudio v3.0 software (Illumina, San Diego, CA, USA). Candidate variants were selected based on a user-defined filtering approach including 24 genes associated with cardiac ion channels and ion homeostasis (Table 2) and high-quality calling (PASS filter). Sequence variants passing the aforementioned filters were analyzed individually, the synonymous ones being discarded. Allele frequency (AF) was obtained from the 1000 genomes project (GRCh37 reference assembly) and Genome Aggregation Database (gnomAD v2.1.1 dataset aligned against the GRCh37 reference) (accessed on 1 April 2023). The following thresholds were considered to define variants in terms of population prevalence: AF above 5% for common variants, AF between 1 and 5% for low-frequency variants, while variants having AF below 1% were categorized as rare variants.

### 4.4. Echocardiographic Image Acquisition and Analysis

All subjects underwent a comprehensive 2D-transthoracic echocardiography study as previously reported in detail [67]. Echocardiography examinations were performed on VIVID E9 (GE Healthcare, Chicago, IL, USA) with a 3.5 MHz array probe, and images were analyzed offline on EchoPac, version BT13. Dedicated acquisitions in apical two and four-chamber views were acquired for left atrium (LA) measurement with a frame rate between 50 and 70 frames/s and at least three heartbeats. Conventional echocardiographic measurements were achieved according to current guidelines [68]. Left ventricular ejection fraction (LVEF) was quantified employing Simpson’s biplane method. The LA diameter was evaluated in parasternal long-axis view in the end systole, while for the two-dimensional LA volumetric analysis, LA volumes were calculated as the average from measurement in apical two and four chamber views using the disk summation algorithm. The maximal volume (LAVmax) was measured just before mitral valve opening, LA minimum volume (LAVmin) at mitral valve closure, and LA pre-A-wave volume (LAVpre-A), just one frame before atrial contraction in accordance with the American Society of Echocardiography and European Association of Cardiovascular Imaging guidelines for chamber quantification [69]. Using the above volumes, LA total (LATEV), passive (LAPEV) and active emptying volumes (LAAEV) as well as fractions (LATEF, LAPEF, LAEI and LAAEF) were calculated as shown in a previous paper [67]. All obtained volumes were indexed to the body surface area. The LA stiffness index (LASI) was defined as E/e′m divided by LASr [70], where E is the mitral E wave velocity and e’m is the average between early-diastolic septal (e’s) and lateral (e’l) mitral annular velocities using tissue Doppler imaging (e’m = e’l + e’s)/2).

The analysis of LA function via speckle tracking was performed in accordance with the guidelines provided by the European Association of Cardiovascular Imaging [71]. LA S and SR were calculated as the average value of apical 4- and 2-C views. The endocardial border of LA was manually tracked so that the region of interest followed the LA endocardial border throughout the cardiac cycle in apical four and two-chamber views. Time-strain and time-strain rate plots were automatically created by the system. On the time-strain plot, we measured the LA strain reservoir (LASr) representing the difference between the strain value at the mitral valve opening and left-ventricular end-diastole (LVED), LA conduit strain (LAScd) as the difference between the strain at onset of atrial contraction and mitral valve opening, and LA contractile strain (LASct) representing the difference between the strain value at LVED and onset of atrial contraction. The zero-strain reference was set at LVED. On the time-strain rate plot, we measured the following: LA reservoir SR as peak systolic positive value (pLASRr), LA conduit SR as the early diastolic negative peak (pLASRcd) and LA contractile SR (pLASRct) as the late diastolic negative peak.

### 4.5. Statistical Analysis

All analyses were completed using the statistical software program SPSS version 23. Data were presented as mean ± SD for continuous variables and as number and percentage for categorical counterparts. Differences between groups if normally distributed were compared with Student’s *t*-test or chi-square, while for non-parametric variables, a Mann–Whitney U test was used. *p*-values were two-tailed, and a cut-off of less than 0.05 was considered statistically significant.

## 5. Conclusions

In patients with HCM and AFib, comparative to genotype-negative subjects, the rs1805127 variant was linked to increased LA remodeling, while the occurrence of the rs55742440 allele was accompanied by a smaller increase in LA size, suggesting that the variation in ion channel genes may exert either a harmful or a protective effect.

## Figures and Tables

**Table 1:** General and echocardiographic characteristics of study cohort illustrated comparatively for AFib+ and AFib− subjects.

Variable	AFib+ (N = 25)	AFib– (N = 20)	*p*-Value
Age at inclusion, years	51.67 ± 14.55	46.35 ± 15	0.27
Male gender, N (%)	20 (80%)	15 (75%)	0.73
History of SCD, N (%)	10 (40%)	4 (20%)	0.27
ICD, N (%)	3 (12%)	4 (20%)	0.43
5-years SCD risk score	5.49 ± 3.92%	2.83 ± 1.37%	0.044
Echocardiography			
Maron 1	3 (12%)	4 (20%)	
Maron 2	3 (12%)	2 (10%)	0.43
Maron 3	19 (76%)	13 (65%)	
Maron 4	0	1 (5%)	
LV maximal wall thickness, mm	20.19 ± 3.45	20.9 ± 6.33	0.65
LV mass indexed, g/m^2^	142.5 ± 44.11	119.52 ± 41.37	0.15
LVEDD, cm	41 ± 7.33	41 ± 9.2	0.99
LVESD, cm	25.7 ± 9.2	24.8 ± 10.79	0.79
LVEDV, ml	106.4 ± 35.21	119.15 ± 45.96	0.37
LVESV, ml	56 ± 31.81	61.84 ± 38.81	0.64
LVEF, (%)	51.6 ± 13.96	52.53 ± 12.18	0.83
LAD, mm	41.61 ± 6.2	39.4 ± 7.62	0.34
Volumetric variables			
LAVmax (mL/m^2^)	38.47 ± 13.7	28.67 ± 9.27	0.007
LAVmin (mL/m^2^)	19 ± 6.8	17 ±4.6	0.247
LAVpre-A (mL/m^2^)	24 ± 2.3	20 ± 7.4	0.029
LATEF (%)	54 ± 10.3	60 ± 8.8	0.041
LAEI (%)	167 ± 60.3	172 ± 50.4	0.763
LAPEF (%)	26 ± 6	25 ± 3.9	0.504
LAAEF (%)	41 ± 12.6	46 ± 14.5	0.231
LAEF (kdyne)	10 ± 5.7	11.6 ± 8	0.456
Deformation variables			
LASr (%)	21 ± 5.6	25 ± 4	0.008
pLASRr (s^−1^)	0.8 ± 0.4	1.2 ± 0.67	0.025
LASI	1.8 ± 0.4	1.4 ± 0.8	0.036
LAScd (%)	17 ± 3.5	18 ± 9.5	0.659
pLASRcd (s^−1^)	−0.4 ± 0.2	−0.6 ± 1.1	0.432
LASct (%)	10 ± 1.2	10.5 ± 4.5	0.634
pLASRct (s^−1^)	−1.3 ± 0.6	−1.4 ± 0.4	0.508

ICD, implantable cardiac defibrillator; LAD, left atrial diameter; LAAEF, left-atrial active ejection fraction; LAEF, left-atrial ejection force; LAEI, left-atrial ejection index; LAPEF, left-atrial passive ejection fraction; LAScd, left-atrial strain during conduit phase; LASct, left-atrial strain during contractile phase; LASI, left-atrial stiffness index; LASr, left-atrial strain during reservoir phase; pLASRr, peak strain rate during reservoir phase; pLASRcd, peak strain rate during conduit phase; pLASRct, peak strain rate during contractile phase; LATEF, left-atrial total ejection fraction; LAVmax, left-atrial volume during systole; LAVmin, left-atrial volume during diastole; LAVpre-A, left-atrial volume before P-wave; LV, left ventricle; LVEDD, left ventricle end-diastolic diameter; LVEDV, left ventricle end-diastolic volume; LVEF, left ventricle ejection fraction; LVESD, left ventricle end-systolic diameter; LVESV, left ventricle end-systolic volume; SCD sudden cardiac death.

**Table 2:** List of the 24 genes analyzed in our study, encoding proteins and references that relate these with atrial fibrillation.

Potassium Channel-Related Genes	Encoding Protein (UniProt ID)	References
*ABCC9*	ATP-binding cassette subfamily C member 9 (O60706)	[7]
*HCN4*	Potassium/sodium hyperpolarization-activated cyclic nucleotide gated potassium channel 4 (Q9Y3Q4)	[16]
*KCNA5*	Potassium voltage-gated channel subfamily A member 5 (P22460)	[17]
*KCND3*	Potassium voltage-gated channel subfamily D member 3 (Q9UK17)	[18]
*KCNE1*	Potassium voltage-gated channel subfamily E member 1 (P15382)	[19]
*KCNE2*	Potassium voltage-gated channel subfamily E member 2 (Q9Y6J6)	[20]
*KCNE3*	Potassium voltage-gated channel subfamily E member 3 (Q9Y6H6)	[21]
*KCNH2*	Potassium voltage-gated channel subfamily H member 2 (Q12809)	[22]
*KCNJ2*	Inward rectifier potassium channel 2 (P63252)	[23]
*KCNJ5*	G protein-activated inward rectifier potassium channel 4 (P48544)	[24]
*KCNJ8*	ATP-sensitive inward rectifier potassium channel 8 (Q15842)	[25]
*KCNQ1*	Potassium voltage-gated channel subfamily KQT member 1 (P51787)	[26]
Sodium channel-related genes	Encoding Protein	
*SCN1B*	Sodium channel subunit beta-1 (Q07699)	[27]
*SCN2B*	Sodium channel subunit beta-2 (O60939)	[27]
*SCN3B*	Sodium channel subunit beta-3 (Q9NY72)	[28]
*SCN4B*	Sodium channel subunit beta-4 (Q8IWT1)	[29]
*SCN5A*	Sodium channel protein type 5 subunit alpha (Q14524)	[30]
Calcium channel-/calcium homeostasis-related genes	Encoding Protein	
*CACNA1C*	Voltage-dependent L-type calcium channel subunit alpha-1C (Q13936)	[31]
*CACNA2D1*	Voltage-dependent calcium channel subunit alpha-2/delta-1 (P54289)	[32]
*CACNB2*	Voltage-dependent L-type calcium channel subunit beta-2 (Q08289)	[33]
*CALM1*	Calmodulin-1 (P0DP23)	
*CASQ2*	Calsequestrin-2 (O14958)	[34]
*JPH2*	Junctophilin-2 (Q9BR39)	[35]
*RYR2*	Ryanodine receptor 2 (Q92736)	[36]

**Table 3:** List of pathogenic or likely pathogenic variants in sarcomeric genes identified in the cohort.

Gene	HGVSc	HGVSp	dbSNP ID	ClinVar ID	Carrier Patient
*MYBPC3*	c.3294G>A	p.Trp1098Ter	rs767039057	520341	AFib+
*MYBPC3*	c.772G>A	p.Glu258Lys	rs397516074	42792	AFib+
*MYH7*	c.2389G>A	p.Ala797Thr	rs3218716	42901	AFib+
*MYH7*	c.715G>A	p.Asp239Asn	rs397516264	43100	AFib+
*TNNI3*	c.557G>A	p.Arg186Gln	rs397516357	43395	AFib+
*TPM1*	c.574G>A	p.Glu192Lys	rs199476315	31882	AFib−

**Table 4:** The number of nonsynonymous variants identified in potassium channel-coding genes in the two groups.

Gene	AFib+ (N = 25)	AFib− (N = 20)
*ABCC9*	0	1
*HCN4*	1	3
*KCNA5*	3	1
*KCND3*	0	1
*KCNE1*	20	20
*KCNE2*	0	0
*KCNE3*	0	0
*KCNH2*	14	8
*KCNJ2*	0	0
*KCNJ5*	25	19
*KCNJ8*	0	1
*KCNQ1*	1	2

AFib, atrial fibrillation.

**Table 5:** The number of nonsynonymous variants identified in sodium channel-coding genes in the two groups.

Gene	AFib+ (N = 25)	AFib− (N = 20)
*SCN1B*	31	26
*SCN2B*	0	2
*SCN3B*	0	0
*SCN4B*	0	1
*SCN5A*	15	9

AFib, atrial fibrillation.

**Table 6:** The number of nonsynonymous variants identified in calcium channel-coding genes in the two groups.

Gene	AFib+ (N = 25)	AFib− (N = 20)
*CACNA1C*	77	53
*CACNA2D1*	0	1
*CACNB2*	16	10
*CALM1*	0	0
*CASQ2*	14	12
*JPH2*	6	3
*RYR2*	9	13

AFib, atrial fibrillation.

## Data Availability

The datasets used and analyzed during the current study are available from the corresponding author upon reasonable request.

## Supplementary Materials

The supporting information can be downloaded at https://www.mdpi.com/article/10.3390/ijms242417244/s1. References [55,56,57,58,59,60,61,62,63,64,65,66,67,68,69,70,71,72,73,74,75,76,77,78] are cited in the Supplementary Materials.
