# Supplementary material for: Impact of rs1805127 and rs55742440 Variants on Atrial Remodeling in Hypertrophic Cardiomyopathy Patients with Atrial Fibrillation: A Romanian Cohort Study
Source: Int J Mol Sci. 2023 Dec 8;24(24):17244. doi: 10.3390/ijms242417244 (PMC10743528; doi:10.3390/ijms242417244)
Supplement: Supplementary file 1 [file ijms-24-17244-s001.zip › ijms-2684600-supplementary.pdf]

Table S1. List of all variants identified in our cohort; variants not found in gnomAD v4 are represented in italic letters; ClinVar likely pathogenic/pathogenic variants are represented in bold letters.

Gene	Genotype	HGVSc	HGVSp	dbSNP ID	AF gnomAD v4*	ClinVar Significance*	No of patients (AFib+ patients)	Reference
ABCC9	*het*	*c.4180C>T*	*p.Gln1394Ter*	*NA*	*NA*	*NA*	*1 (0)*	
HCN4	het	c.107G>A	p.Gly36Glu	rs143090627	0.06426	B/LB	4 (2)	
KCNA5	het	c.751G>A	p.Ala251Thr	rs12720442	0.01349	B/LB	1 (1)	
KCNA5	het	c.919C>T	p.Pro307Ser	rs17215409	0.003302	CON (VUS/LB)	1 (1)	
KCNA5	het	c.1150G>A	p.Gly384Arg	rs76708779	0.001198	B/LB	2 (1)	
KCND3	*het*	*c.310C>T*	*p.His104Tyr*	*NA*	*NA*	*NA*	*1 (0)*	
KCNE1	het	c.112A>G	p.Ser38Gly	rs1805127	0.0006525	B	40 (25)	[55-60]
KCNH2	*het*	*c.533C>T*	*p.Ser178Leu*	*NA*	*NA*	*NA*	*1 (0)*	
KCNH2	*het*	*c.916+1G>T*		*NA*	*NA*	*NA*	*1 (0)*	
KCNH2	hom	c.2690A>C	p.Lys897Thr	rs1805123	0.3125	B	15 (7)	[72]
KCNH2	**het**	**c.3007G>T**	**p.Asp1003Tyr**	**rs794728402**	**7.02E-07**	**LP**	**1 (0)**	
KCNH2	het	c.3140G>T	p.Arg1047Leu	rs36210421	0.02106	CON (VUS/B/LB)	2 (2)	
KCNH2	het	c.3215C>T	p.Thr1072Met	rs781624566	0.00001242	VUS	1 (0)	
KCNH2	*het*	*c.3241G>T*	*p.Val1081Leu*	*NA*	*NA*	*NA*	*1 (0)*	
KCNJ5	*het*	*c.759C>A*	*p.Asn253Lys*	*rs772933591*	*0.000008212*	*NA*	*1 (1)*	
KCNJ5	hom	c.844C>G	p.Gln282Glu	rs7102584	0.9846	B	43 (25)	
KCNJ8	*het*	*c.68G>T*	*p.Arg23Leu*	*NA*	*NA*	*NA*	*1 (1)*	
KCNQ1	het	c.181C>A	p.Pro61Thr	rs1273257287	0.00001611	VUS	1 (1)	
KCNQ1	het	c.1343C>T	p.Pro448Leu	rs12720449	0.00003967	CON (VUS/LB)	2 (0)	[58, 73-74]
SCN1B	het	c.412G>A	p.Val138Ile	rs72558029	0.005004	B/LB	1 (0)	
SCN1B	het	c.629T>C	p.Leu210Pro	rs55742440	0.3703	B	27 (16)	
SCN1B	het	c.744C>A	p.Ser248Arg	rs67701503	0.1438	B	14 (8)	
SCN1B	het	c.749G>C	p.Arg250Thr	rs67486287	0.0003606	B	14 (8)	
SCN1B	het	c.769G>A	p.Gly257Arg	rs72558028	0.002166	B/LB	1 (0)	[75]
SCN2B	*het*	*c.400G>C*	*p.Asp134His*	*NA*	*NA*	*NA*	*1 (1)*	

SCN2B	*het*	*c.503delG*	*p.Gly168AlafsTer10*	*NA*	*NA*	*NA*	*1 (0)*	
SCN4B	het	c.613T>C	p.Ser205Pro	rs761183228	0.00002541	VUS	1 (0)	
SCN5A	**het**	**c.393-1C>T**		**rs759235726**	**0.000006229**	**CON (LP/VUS)**	**1 (1)**	
SCN5A	het	c.1336G>A	p.Glu446Lys	rs199473339	0.0002908	CON (VUS/B/LB)	1 (0)	[76]
SCN5A	het	c.1673A>G	p.His558Arg	rs1805124	0.291	B/LB	20 (12)	[77-78]
SCN5A	**het**	**c.2431C>T**	**p.Arg811Cys**	**rs794728864**	**0.000005584**	**CON (LP/VUS)**	**1 (0)**	
SCN5A	*het*	*c.3118G>T*	*p.Gly1040Trp*	*NA*	*NA*	*NA*	*1 (1)*	
CACNA1C	*het*	*c.1412C>A*	*p.Thr471Asn*	*rs1599718556*	*NA*	*VUS*	*1 (0)*	
CACNA1C	*het*	*c.1635C>A*	*p.His545Gln*	*NA*	*NA*	*NA*	*1 (1)*	
CACNA1C	*het*	*c.2084C>A*	*p.Ser695Tyr*	*NA*	*NA*	*NA*	*1 (1)*	
CACNA1C	*het*	*c.326T>C*	*p.Leu109Pro*	*NA*	*NA*	*NA*	*1 (1)*	
CACNA1C	*het*	*c.4502T>C*	*p.Val1501Ala*	*NA*	*NA*	*NA*	*1 (0)*	
CACNA1C	het	c.5294C>G	p.Ala1765Gly	rs201492706	0.0005878	CON (VUS/B/LB)	1 (1)	
CACNA1C	hom	c.5603C>T	p.Pro1868Leu	rs10848683	0.7956	B	38 (21)	
CACNA1C	hom	c.5605A>G	p.Met1869Val	rs10774053	0.8199	B	38 (21)	
CACNA1C	hom	c.5678A>G	p.Lys1893Arg	rs10774054	0.9999	B	39 (21)	
CACNA1C	het	c.5842G>A	p.Glu1948Lys	rs200231105	0.0004549	CON (VUS/ B/LB)	3 (2)	
CACNA1C	het	c.5858C>T	p.Thr1953Met	rs201777030	0.001114	B/LB	4 (2)	
CACNA1C	*het*	*c.6113C>A*	*p.Pro2038Gln*	*NA*	*NA*	*NA*	*1 (0)*	
CACNA1C	het	c.6167G>A	p.Arg2056Gln	rs112414325	0.002302	B/LB	1 (1)	
CACNA2D1	*het*	*c.1300A>G*	*p.Met434Val*	*rs199961189*	*0.000006225*	*NA*	*1 (1)*	
CACNB2	*het*	*c.91C>A*	*p.Pro31Thr*	*rs766827150*	*0.000002756*	*NA*	*1 (0)*	
CACNB2	*het*	*c.465C>A*	*p.Asn155Lys*	*NA*	*NA*	*NA*	*1 (1)*	
CACNB2	het	c.641G>C	p.Ser214Thr	rs149253719	0.001059	CON (VUS/ B/LB)	1 (0)	
CACNB2	*het*	*c.808G>T*	*p.Glu270Ter*	*NA*	*0.000001591*	*NA*	*1 (1)*	
CACNB2	*het*	*c.1196C>A*	*p.Ser399Tyr*	*NA*	*NA*	*NA*	*1 (1)*	
CACNB2	*het*	*c.1661C>A*	*p.Thr554Lys*	*NA*	*NA*	*NA*	*1 (1)*	
CACNB2	het	c.1965T>G	p.Asp655Glu	rs58225473	0.1612	B	20 (13)	
CASQ2	het	c.196A>G	p.Thr66Ala	rs4074536	0.3076	B	26 (15)	
JPH2	*het*	*c.1039G>T*	*p.Val347Phe*	*NA*	*NA*	*NA*	*1 (0)*	

JPH2	hom	c.1186G>A	p.Ala396Thr	rs3810510	0.1512	B	7 (5)	
JPH2	het	c.1513G>A	p.Gly505Ser	rs140740776	0.006232	B/LB	1 (0)	
RYR2	*het*	*c.29A>C*	*p.Glu10Ala*	*NA*	*NA*	*VUS*	*1 (1)*	
RYR2	*het*	*c.3944C>A*	*p.Ser1315Tyr*	*NA*	*NA*	*NA*	*1 (0)*	
RYR2	het	c.4198A>G	p.Ser1400Gly	rs56229512	0.02153	B/LB	1 (1)	
RYR2	*het*	*c.4552C>A*	*p.Leu1518Ile*	*NA*	*NA*	*NA*	*1 (1)*	
RYR2	het	c.5056C>T	p.Leu1686Phe	rs752684338	0.000009914	VUS	1 (1)	
RYR2	het	c.5656G>A	p.Gly1886Ser	rs3766871	0.03743	B/LB	1 (0)	
RYR2	het	c.8873A>G	p.Gln2958Arg	rs34967813	0.2643	B/LB	16 (7)	

AF allele frequency; AFib atrial fibrillation; B benign; CON variant with conflicting interpretations of pathogenicity; LB likely benign; LP likely pathogenic; NA data not available; P pathogenic; VUS variant of uncertain significance.

* accessed on 15 November 2023

References

55. Fatini, C.; Sticchi, E.; Genuardi, M.; Sofi, F.; Gensini, F.; Gori, A.M.; Lenti, M.; Michelucci, A.; Abbate, R.; Gensini, G.F. Analysis of minK and eNOS genes as candidate loci for predisposition to non-valvular atrial fibrillation. Eur. Heart J. 2006, 27, 1712–1718. https://doi.org/10.1093/eurheartj/ehl087.

56. Prystupa, A.; Dzida, G.; Myśliński, W.; Małaj, G.; Lorenc, T. MinK gene polymorphism in the pathogenesis of lone atrial fi-brillation. Kardiol. Pol. 2006, 64, 1205–1211.

57. Yao, J.; Ma, Y.T.; Xie, X.; Liu, F.; Chen, B.D. Association of KCNE1 genetic polymorphisms with atrial fibrillation in a Chinese Han population. Genet. Test. Mol. Biomark. 2012, 16, 1343–1346. https://doi.org/10.1089/gtmb.2012.0149.

58. Zeng, Z.; Tan, C.; Teng, S.; Chen, J.; Su, S.; Zhou, X.; Wang, F.; Zhang, S.; Gu, D.; Makielski, J.C.; et al. The single nucleotide pol-ymorphisms of IKs potassium channel genes and their association with atrial fibrillation in a Chinese population. Cardiology 2007, 108, 97–103. https://doi.org/10.1159/000095943.

59. Xu, L.X.; Yang, W.Y.; Zhang, H.Q.; Tao, Z.H.; Duan, C.C. Study on the correlation between CETP TaqIB, KCNE1 S38G and eNOS T-786C gene polymorphisms for predisposition and non-valvular atrial fibrillation. Chin. J. Epidemiol. 2008, 29, 486–492.

60. Liang, C.; Li, X.; Xu, Y.; Chen, Q.; Wu, Y.; Wang, W.; Li, W.; Qiu, M. KCNE1 rs1805127 Polymorphism Increases the Risk of Atrial Fibrillation: A Meta-Analysis of 10 Studies. PLoS ONE 2013, 8, e68690. https://doi.org/10.1371/journal.pone.0068690.

72. Smith JG, Almgren P, Engström G, Hedblad B, Platonov PG, Newton-Cheh C, Melander O. Genetic polymorphisms for estimating risk of atrial fibrillation: a literature-based meta-analysis. J Intern Med. 2012 Dec;272(6):573-82. doi: 10.1111/j.1365-2796.2012.02563.x. Epub 2012 Jul 27. PMID: 22690879; PMCID: PMC3763745.

73. Zeng ZY, Pu JL, Tan C, Teng SY, Chen JH, Su SY, Zhou XY, Zhang S, Li YS, Wang FZ, Gu DF. [The association of single nucleotide polymorphism of slow delayed rectifier K+ channel genes with atrial fibrillation in Han nationality Chinese]. Zhonghua Xin Xue Guan Bing Za Zhi. 2005 Nov;33(11):987-91. Chinese. PMID: 16563243.

74. Chen LY, Goh JM, Wong RC, Hsu LF, Foo D, Benditt DG, Ling LH, Heng CK. Comprehensive mutation scanning of KCNQ1 in 111 Han Chinese patients with lone atrial fibrillation. Heart Asia. 2010 Nov 8;2(1):126-8. doi: 10.1136/ha.2010.002832. PMID: 27325960; PMCID: PMC4898533.

75. Husser D, Ueberham L, Hindricks G, Büttner P, Ingram C, Weeke P, Shoemaker MB, Adams V, Arya A, Sommer P, Darbar D, Roden DM, Bollmann A. Rare variants in genes encoding the cardiac sodium channel and associated compounds and their impact on outcome of catheter ablation of atrial fibrillation. PLoS One. 2017 Aug 24;12(8):e0183690. doi: 10.1371/journal.pone.0183690. PMID: 28837624; PMCID: PMC5570360.

76. McNair WP, Sinagra G, Taylor MR, Di Lenarda A, Ferguson DA, Salcedo EE, Slavov D, Zhu X, Caldwell JH, Mestroni L; Familial Cardiomyopathy Registry Research Group. SCN5A mutations associate with arrhythmic dilated cardiomyopathy and commonly localize to the voltage-sensing mechanism. J Am Coll Cardiol. 2011 May 24;57(21):2160-8. doi: 10.1016/j.jacc.2010.09.084. PMID: 21596231; PMCID: PMC9689753.

77. An R, Liu J, Zhang J, Yao F, Tian D, Liang F, Li W, Li D, Wang Y, Yan S, Yang Q, Zhang Y, Su X. Risk factors and SCN5A-H558R polymorphism for atrial fibrillation in Tibetans living at different altitudes. Medicine (Baltimore). 2022 Nov 18;101(46):e31778. doi: 10.1097/MD.0000000000031778. PMID: 36401443; PMCID: PMC9678620.

78. Malakootian M, Jalilian M, Kalayinia S, Hosseini Moghadam M, Heidarali M, Haghjoo M. Whole-exome sequencing reveals a rare missense variant in DTNA in an Iranian pedigree with early-onset atrial fibrillation. BMC Cardiovasc Disord. 2022 Feb 11;22(1):37. doi: 10.1186/s12872-022-02485-0. PMID: 35148685; PMCID: PMC8832862.
